# Research on the True Triaxial Mechanical Properties of Concrete under the Coupling Action of High Temperature and Biaxial Unequal Lateral Pressure

**DOI:** 10.3390/ma15145014

**Published:** 2022-07-19

**Authors:** Biao Ren, Tengjiao Wang, Jinyu Xu, Zhihang Wang, Yan Lv, Yipeng Ning

**Affiliations:** 1 Aviation Engineering School, Air Force Engineering University, Xi’an 710043, China; zhh17713821307@163.com (B.R.); wtengjiao83087@163.com (T.W.); wangzhihangafeu@163.com (Z.W.); lvyanleo30@163.com (Y.L.); ningyipeng@163.com (Y.N.); 2 School of Mechanics, Civil Engineering and Architect, Northwest University of Technology, Xi’an 710064, China

**Keywords:** concrete, triaxial compression, high temperature damage, strength formula, side pressure ratio

## Abstract

Based on engineering practice and practical needs, this paper takes ordinary concrete specimens as the research object, and adopts a high-temperature true triaxial loading test system to carry out high-temperature uniaxial and true triaxial static compression tests of concrete under high-temperature conditions. By comparing with normal temperature conditions, this paper analyzes the influence of the coupling effect of high-temperature and biaxial unequal lateral pressure on the static mechanical properties of concrete. By analyzing the experimental data, we reached a series of conclusions and carried out theoretical research on this basis. High temperatures can significantly affect the uniaxial static pressure strength characteristics, deformation characteristics, and failure mode of concrete. When the temperature exceeds 400 °C, the compressive strength decreases significantly, the peak strain increases sharply, and the plasticity of concrete is further enhanced. The coupling effect of high-temperature deterioration and lateral pressure strengthening makes the true triaxial mechanical properties of concrete change significantly; 0.6:0.2 and 400 °C are the turning points of side pressure ratio and temperature that affect the change law of the true triaxial mechanical properties of concrete, respectively. Based on the study of the high-temperature deterioration factor and lateral pressure strengthening factor, this paper further puts forward a concrete strength formula under the coupling action of high temperature and biaxial unequal lateral pressure. It was verified that the formula has a high accuracy.

## 1. Introduction

As the most common building material, concrete is widely used in both industrial and civil buildings and protection engineering [1]. During service, concrete materials are generally in a normal temperature environment and mainly bear static loads. However, when buildings are on fire and protection works are hit by weapons, concrete is often exposed to a high-temperature environment. High temperature can cause complex physical and chemical reactions in various components in concrete, resulting in significant changes in the mechanical properties of concrete [2,3,4]. In addition, because underground protection engineering bears the pressure of the surrounding rock and the complex stress at the joints of beams, columns, plates, and other components of industrial and civil buildings, concrete is often in a state of biaxial or triaxial stress. With the continuous development of society, people have higher and higher requirements for buildings. They not only need the building to maintain a normal working state under normal temperature and uniaxial compression, but also put forward requirements for the triaxial compression performance of concrete under high temperature. Therefore, it is necessary to study the static true triaxial compressive properties of concrete under high temperature.

In order to evaluate the safety status of buildings after fire and improve the fire resistance of concrete components, researchers from various countries have carried out high-temperature mechanical tests of concrete since the 1960s. The researchers found that the mechanical properties of high-strength concrete deteriorate with the increase of heating temperature. With the increase of heating temperature, the peak stress, peak strain, and elastic modulus of concrete decrease, and the change range is small when the temperature is lower than 400 °C. After the temperature exceeds 400 °C, its properties deteriorate rapidly [5]. In a high-temperature environment, concrete will burst under the coupling action of thermal cracking and pore water (steam) pressure [6]. Some studies have found that the hot compressive strength of SCC (self-compacting concrete) decreases with increasing temperature. Compared with normal-strength SCC, high-strength SCC possesses a larger compressive strength when exposed to high temperature. Another result of the tests is that the addition of polypropylene fibers decreased the strength and probability of explosive spalling [7]. However, it was found that the compressive strength of the concrete increased initially, with an increase in the temperature up to 300 °C; however, with a further increase in the temperature, the compressive strength was found to decrease [8]. In order to study the influence of different fibers on the deformation capacity of cylinder concrete at high temperature, Liu Genjin and others carried out relevant experimental research by burning liquefied gas to simulate the high temperature effect of fire on concrete. The results showed that the internal and external temperature difference and radial displacement of concrete materials reached a maximum at the stage of gas temperature stability [9]. Meng Long and others studied the dynamic splitting tensile properties of concrete at high temperature, with the help of a large-diameter Hopkinson compression bar, and found that the dynamic splitting tensile strength decreased with the increase of temperature [10].

The spatial scale characteristics of concrete structures determine that concrete materials are often, not only subjected to the force in one direction, but are in the state with three forces, which is particularly obvious at the joints of concrete components. By studying the mechanical characteristics of concrete under triaxial stress, this can help to understand the mechanical behavior of concrete components in the actual working environment. Research on the true triaxial static compression mechanical properties of concrete is often transformed into research on the uniaxial static compression mechanical properties of concrete under biaxial unequal stress. In this field, scholars from various countries have carried out a large number of experimental studies and preliminarily established the theoretical basis of concrete triaxial tests.

Through a triaxial static mechanical property test of high-strength concrete, researchers found that the axial compressive strength of high-strength concrete increases with the increase of confining pressure, but compared with ordinary concrete, its strength is less affected by confining pressure [11]. Girgin studied the triaxial compressive mechanical properties of ultra-high strength concrete, and summarized a general formula for calculating the triaxial compressive strength of this concrete [12]. In a study of the influence of water cement ratio on the triaxial compressive properties of ordinary concrete, it was found that the mechanical properties of concrete are little affected by water cement ratio under the constraint of high confining pressure [13]. While, loading path and confining pressure conditions can significantly affect the mechanical properties of concrete [14,15]. In terms of concrete failure mode, researchers found that confining pressure can significantly improve the strength characteristics of concrete, and with the increase of confining pressure, concrete changes from obvious brittle failure to significant ductile failure [16]. Researchers have established a concrete triaxial compression strength calculation model related to the mix proportion, and found that the model can effectively calculate the ultimate strength of concrete under triaxial compression [17]. Based on the three-dimensional discrete element method, Tran studied the mechanical behavior of concrete under high lateral pressure. Combined with the uniaxial and triaxial compression test data, the constitutive equation of concrete under triaxial compression was derived, which can be used to predict the mechanical response of concrete under different constraints [18]. Yu found that the strength and peak strain of reactive powder concrete under biaxial and triaxial stress were significantly higher than those under uniaxial stress. When the ratio of the second principal stress to the third principal stress was 0.25, the triaxial strength of reactive powder concrete reached the maximum [19,20]. Li Jing used a true triaxial loading test system to carry out a static compression test of cubic concrete specimens. The results showed that the strain changes of the *x*-axis and *z*-axis were linear during the loading process [21]. In 2021, in order to establish a compression constitutive model of lightweight aggregate concrete, researchers carried out a true triaxial compression test of concrete. It was found that, under the condition of a high lateral pressure ratio, the intermediate principal stress had the most obvious effect on the triaxial compressive strength of concrete [22].

It can be seen from the above that, at present, research on the mechanical properties of concrete at high temperature mainly focuses on the mechanical properties of uniaxial static compression at high temperature, while research on the mechanical properties of concrete under true triaxial static compression at high temperature is relatively scarce. With fire and deep-underground environments, concrete members are often in the coupling state of high temperature and three-dimensional complex stress. Therefore, it is necessary to study the high-temperature true triaxial mechanical properties of concrete.

In this paper, a high-temperature true triaxial loading test system was used to carry out high-temperature uniaxial and true triaxial static compression tests of concrete. By comparing the mechanical properties of concrete under uniaxial static compression at high temperature, the effects of different temperature levels and different lateral pressure ratios on the strength characteristics, deformation characteristics, and failure modes of concrete under high-temperature true triaxial static compression were systematically analyzed. Combined with the test results and relevant research theories, the internal influence mechanism was analyzed, and a concrete strength formula under the coupling action of high temperature and biaxial unequal lateral pressure is put forward.

## 2. Materials and Tests

### 2.1. Test Material

In this paper, the specimen was poured with ordinary concrete, and the raw materials required for its preparation included: cement, aggregate, water, and water reducer. The cement selected, P.O42.5, was produced by *Xi’an Lantian Yaobai Cement Co., Ltd.*, (*Xi’an, China)* and its specific chemical composition and relevant performance indexes are shown in Table 1 and Table 2, respectively. Limestone crushed stone was used as coarse aggregate, in which large crushed stone accounted for 70% and small crushed stone accounted for 30%. The main technical indexes of the two kinds of crushed stone are shown in Table 3, and the apparent density of the graded coarse aggregate was 2704 kg·m^−3^. The fine aggregate was Bahe medium sand, with an apparent density of 2650 kg/m^3^, bulk density of 1490 kg/m^3^, and mud content of 1.3%. The water used for the preparation of the test piece was tap water, cleaned in the laboratory. JKPCA-02 polycarboxylic acid high-performance water reducer produced by *Shaanxi Jingcheng building materials Co., Ltd.* (*Xi’an, China)* were used to improve the workability of the mixed concrete. The water reduction rate was 26%, and the gas content was 2.8%.

### 2.2. Specimen Preparation and Test Equipment

The mix proportion of concrete required for this test was designed according to the provisions of *Code for Mix Proportion Design of Ordinary Concrete (JGJ 55-2011)* [23]. After calculation, it was determined that the water cement ratio of mixed concrete was 0.43, the sand ratio was 34%, and the dosage of water reducing agent was 1%. The mix of concrete used in the test is shown in Table 4.

**Figure 1:**
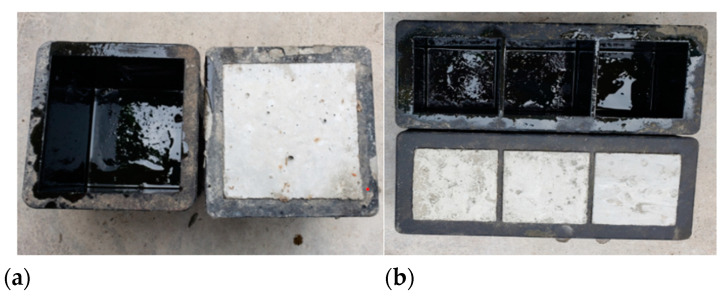
Test pieces of two sizes. (**a**) 150 mm × 150 mm × 150 mm cube. (**b**) 70.7 mm × 70.7 mm × 70.7 mm cube.

According to *GB/T 50081-2019 Test Method Standard for Physical and Mechanical Properties of Concrete* [24] and test requirements, two concrete specimens of different specifications needed to be poured in this paper. One was a 150 mm × 150 mm × 150 mm cube specimen for a standard compressive strength test of concrete, and another 70.7 mm × 70.7 mm ×70.7 mm cube specimen was used to test the static mechanical properties of concrete under the coupling action of high temperature and biaxial unequal lateral pressure. The two test pieces are shown in Figure 1. The specimens prepared according to the specification were cured under standard conditions for 28 days, to obtain the 28 d age specimens required for the test.

**Figure 2:**
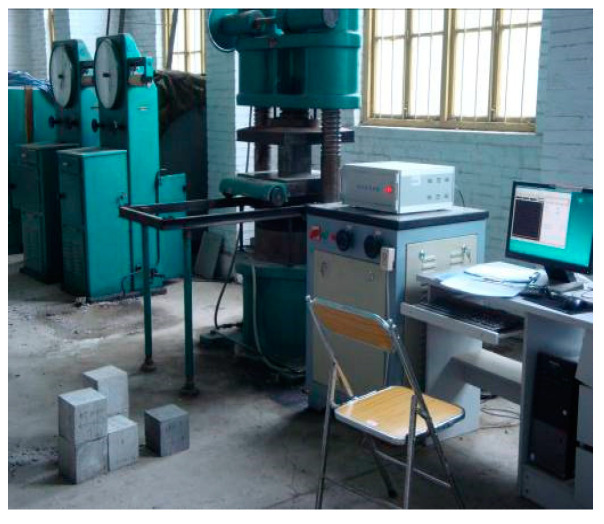
HYY electro-hydraulic servo material test system.

**Figure 3:**
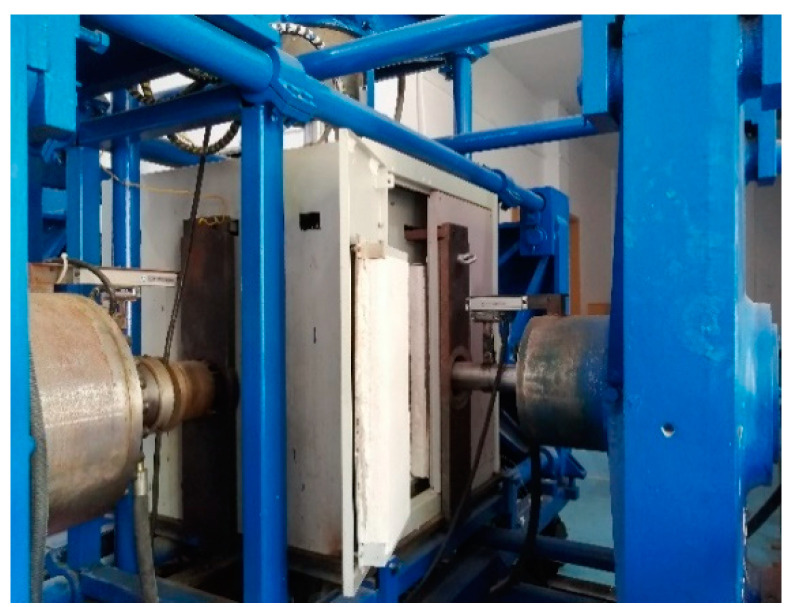
High-temperature true triaxial loading test system.

In this paper, a HYY electro-hydraulic servo material test system was used to carry out a uniaxial static compression test of concrete at room temperature, as shown in Figure 2, and a high-temperature true triaxial loading test system was used to carry out high-temperature uniaxial and true triaxial static compression tests of concrete. The high-temperature uniaxial and true triaxial static compression tests of concrete were completed on a high-temperature true triaxial loading test system. The test system was designed and developed by *Luoyang tengyang Machinery Technology Co., Ltd.*, (Luoyang, China) and the applicable specimen size is a 70.7 mm × 70.7 mm × 70.7 mm cube. The test system is divided into a high-temperature heating system, static loading system, dynamic loading system, and data acquisition system. It can be used for true triaxial static compression tests of concrete, rock, and other materials at room temperature and high temperature, as shown in Figure 3.

### 2.3. Test Scheme

In this paper, according to the *Standard for Test Methods of Physical and Mechanical Properties of Concrete* [24] *(GB/T 50081-2019)*, a HYY electro-hydraulic servo material test system was used to carry out uniaxial static compression tests at room temperature on 150 mm × 150 mm × 150 mm cube concrete specimens aged 28 days, and the test data were recorded. The main purpose of this test was to calibrate the concrete strength, to check whether the concrete strength met the requirements. 

The high-temperature true triaxial loading test system was used to carry out uniaxial static compression tests on 70.7 mm × 70.7 mm × 70.7 mm cube concrete specimens, at five temperature levels (20 °C, 200 °C, 400 °C, 600 °C, and 800 °C), and the test data were recorded. The high-temperature true triaxial loading test system was used to conduct high-temperature true triaxial static compression tests on 70.7 mm × 70.7 mm × 70.7 mm cube concrete specimens. The equipment can apply a constant lateral pressure to the concrete specimen in the Z and X directions using a hydraulic device. The lateral pressure ratio is the ratio of the lateral pressure applied in the Z and X directions. The greater the lateral pressure ratio, the greater the lateral pressure in the Z-direction. Four different side pressure ratios were set, and five temperature grades (20 °C, 200 °C, 400 °C, 600 °C, and 800 °C) were set under each side pressure ratio. 

**Figure 4:**
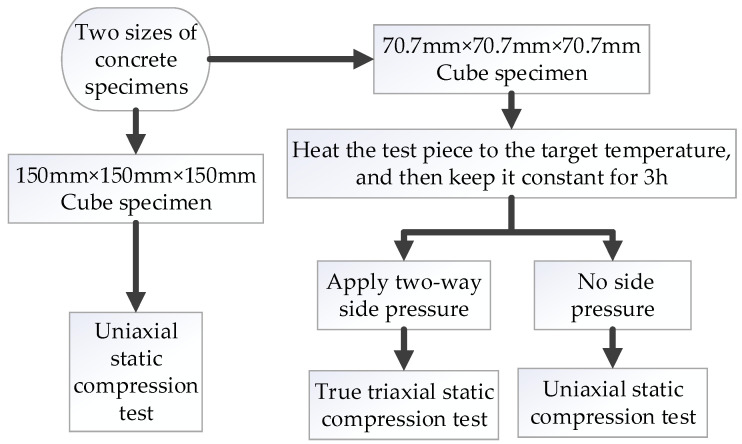
Test flow chart.

See Table 5 for the setting of lateral pressure. “Z:X = 0.4:0.2” in the table means that the lateral pressure of Z direction and X direction was 0.4 and 0.2 times the uniaxial compressive strength of concrete at this temperature. *σ_x_* and *σ_z_* represent the specific values of the side pressure, and the side pressure settings of the other test groups were similar. In this paper, *σ_t_* is defined as the uniaxial compressive strength of concrete at the corresponding temperature. For example, “the lateral pressure in Z direction is 0.4*σ_t_*“ means that the lateral pressure in the Z direction is equal to 0.4 times the uniaxial compressive strength at this test temperature. The average value of three test data was taken as the test result for each group of tests above. The specific test process is shown in Figure 4.

## 3. Analysis of Test Results

The true triaxial static compressive strength of concrete is essentially the peak stress reached by the compressive stress σ_y_ in the Y direction of a concrete specimen under the condition of constant lateral compressive stress σ_z_ and σ_x_. Static compressive strength is an important index to characterize the static compressive strength characteristics of concrete under the condition of true triaxial loading. In the actual service environment, concrete is often in complex stress conditions, and different stress conditions have different effects on the true triaxial static compressive strength of concrete. Therefore, this paper studied the true triaxial static compressive strength of concrete under four side pressure ratios at each temperature level.

The results of the high-temperature uniaxial static compression test and high-temperature true triaxial static compression test of concrete are shown in Table 6 and Table 7.

### 3.1. Strength Characteristic Analysis

The 150 mm × 150 mm × 150 mm cube concrete specimen poured in the test was tested for compressive strength, which was used to determine the cubic compressive strength of the concrete, and did not participate in the subsequent test. The strength of the cube concrete specimens with an uniaxial compressive strength of 70.7 mm × 70.7 mm × 70.7 mm described in this paper was measured using uniaxial compression in a high-temperature true triaxial loading test system. In order to facilitate the research, the concrete strength retention rate *f_g_* was introduced, and its expression is shown in Formula (1).

(1) $$f_{g}=\frac{\sigma_{y}}{\sigma_{0}}\times 100\%$$

where *σ_y_* is the true triaxial static compressive strength, and *σ_0_* is the uniaxial compressive strength at room temperature.

**Figure 5:**
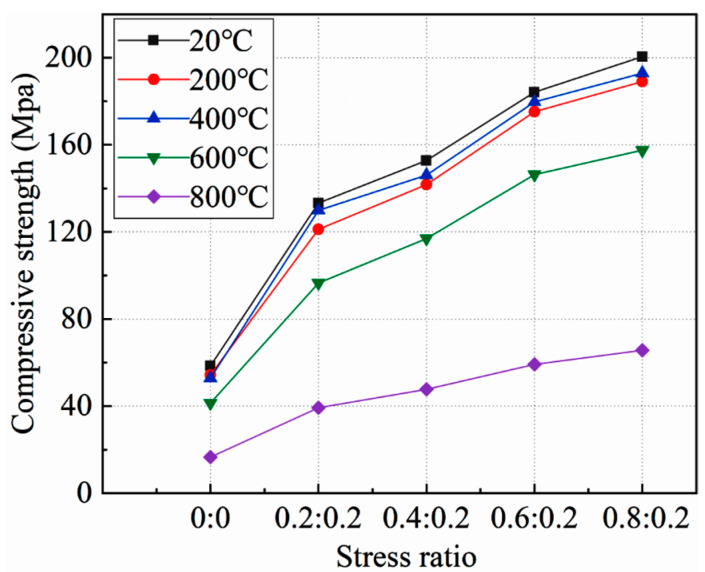
Relationship between static compressive strength and side pressure ratio of concrete.

Figure 5 shows the relationship between the static compressive strength of concrete under high temperature and the side pressure ratio. In order to facilitate the description, the “side pressure ratio is 0:0” was used to represent the uniaxial compression test.

As can be seen from the figure:

At the same temperature, the true triaxial static compressive strength of concrete was significantly higher than that of the uniaxial static compressive strength, and increased with the increase of the side pressure ratio. When the side pressure ratio was 0.8:0.2, the true triaxial static compressive strength of concrete at 20 °C~800 °C reached the maximum, which was 243.09%, 248.30%, 239.79%, 280.45%, and 295.53% higher than the uniaxial static compressive strength at each test temperature. This shows that lateral pressure can significantly improve the true triaxial static compressive strength of concrete, and the influence law of lateral pressure on the compressive strength of concrete at different temperatures is similar. At the same temperature, with the increase of side pressure ratio, the true triaxial static compressive strength of concrete did not show a linear growth trend, and its growth rate was first large, then small, and then large. 

**Figure 6:**
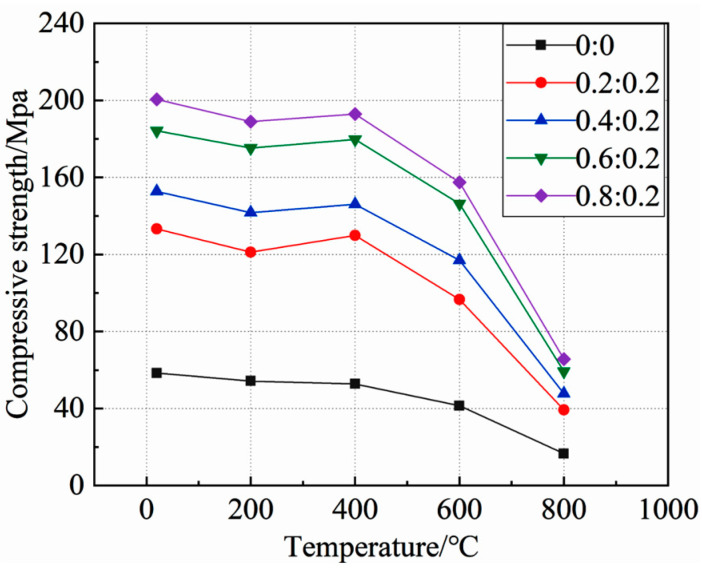
Relationship between concrete static compressive strength and temperature.

Figure 6 shows the relationship between the static compressive strength and temperature of concrete under different side pressure ratios.

It can be seen from the figure that under the same side pressure ratio, the static compressive strength of concrete decreased with the increase of temperature. When the temperature was 20~400 °C, the overall change range of concrete compressive strength was small. At 400 °C, the strength was slightly higher than that at 200 °C, but still lower than that at normal temperature, indicating that when the temperature was lower than 400 °C, the static compressive strength of concrete was less affected by temperature. When the temperature exceeded 400 °C, the static compressive strength of the concrete decreased sharply, and reached the minimum value at 800 °C. Under the condition of 800 °C, the compressive strength of concrete with each side pressure ratio only accounted for 28.41%, 29.45%, 31.27%, 32.16%, and 32.75% of the compressive strength under normal temperature, indicating that a high temperature, above 400 °C, can significantly reduce the static compressive strength of concrete, and that the effect on concrete under different side pressure ratios is similar.

**Figure 7:**
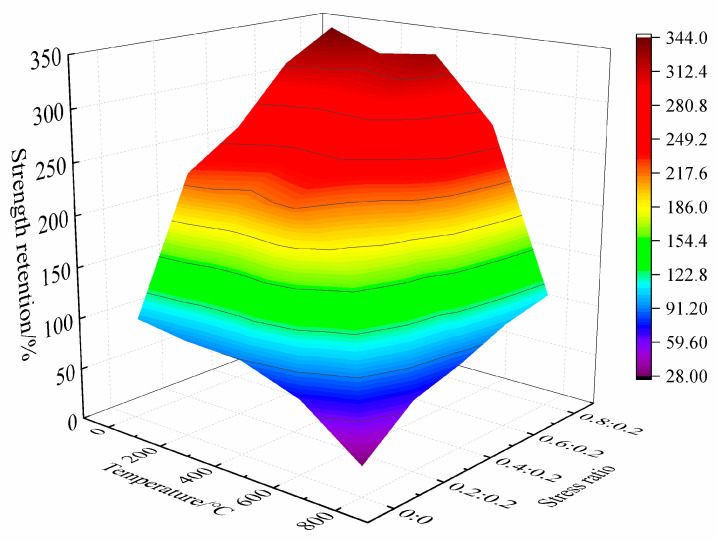
Strength retention rate of concrete under the coupling action of high temperature and biaxial unequal lateral pressure.

Figure 7 shows the strength retention rate of concrete under the coupling action of high temperature and a biaxial unequal lateral pressure. 

As can be seen from the figure:

Under each test condition, the strength retention rate of the concrete changed greatly. The lower the temperature and the greater the side pressure ratio, the greater the strength retention rate of the concrete and, on the contrary, the smaller the strength retention rate. According to the strength retention rate, the influence of the coupling action of high temperature and biaxial unequal lateral pressure on the strength characteristics of concrete can be divided into three stages: saturation stage, strengthening stage, and deterioration stage. In the saturation stage, when the temperature does not exceed 400 °C and the side pressure ratio is greater than or equal to 0.6:0.2, the strength retention rate of concrete fluctuates slightly, but basically remains above 300%; indicating that, in this case, the temperature has little effect on the compressive strength of concrete, and the strengthening effect of side pressure tends to be saturated. In the strengthening stage, when the temperature is 400 °C~600 °C and the side pressure ratio is less than 0.6:0.2, the strength retention rate of concrete is still greater than 100%, and the increase of strength retention rate is larger with an increase of side pressure ratio; indicating that at this time, temperature has a certain deterioration effect on the compressive strength of concrete, but the side pressure effect is dominant. In the deterioration stage, when the temperature exceeds 600 °C, the strength retention rate of concrete decreases sharply. Increasing the lateral pressure ratio has little effect on the strength retention rate of concrete, indicating that temperature degradation is dominant and the reinforcement effect of lateral pressure is hidden.

In conclusion, the coupling effect of high temperature and biaxial unequal lateral pressure can significantly affect the strength characteristics of concrete, but the dominant position of temperature deterioration and lateral pressure strengthening is different in the coupling range of different temperatures and lateral pressure ratios.

### 3.2. Strength Characteristic Analysis

The strain of concrete during compression failure is an important factor reflecting the mechanical properties of concrete. Therefore, it is necessary to explore the effects of temperature and side pressure ratio on the deformation characteristics of concrete under triaxial compression. Among these, the strain characteristics in the failure direction of principal pressure are the focus of research. Similarly, in order to facilitate this research, the concrete strength retention rate *f_s_* is introduced, and its expression is shown in Formula (2).

(2) $$f_{s}=\frac{\varepsilon_{y}}{\varepsilon_{p}}$$

In the formula, *ε_y_* is the peak compressive strain of concrete in Y direction, and *ε*_0_ is the uniaxial static pressure peak strain at room temperature.

**Figure 8:**
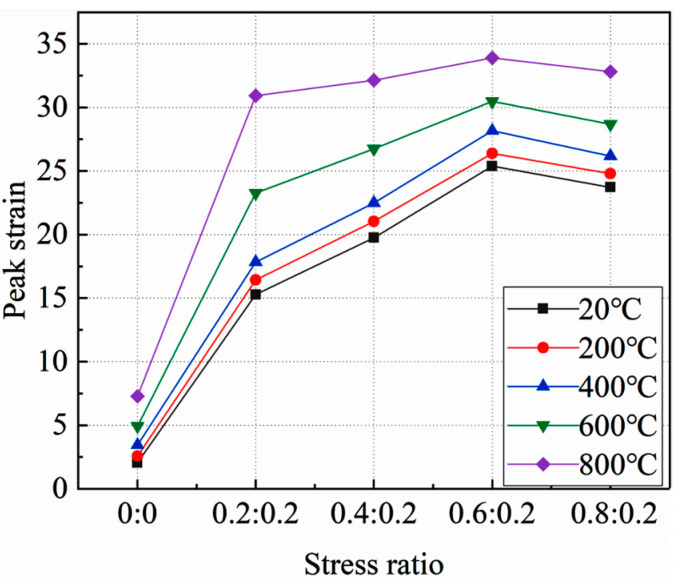
Relationship between peak strain of concrete static pressure and side pressure ratio.

The relationship between the peak static pressure strain of concrete under high temperature and the side pressure ratio is shown in Figure 8.

As can be seen from the figure:

At the same temperature, the peak strain of the true triaxial static pressure of concrete is significantly higher than that of the uniaxial static pressure. With the increase of lateral pressure ratio, the peak strain of concrete first increases and then decreases. When the side pressure ratio is 0.6:0.2, the peak strain reaches the maximum value, which increases by 113.83%, 926.4%, 718.65%, 518.26%, and 365.53% compared with the peak strain of concrete under uniaxial compression at various temperatures, indicating that a bidirectional side pressure can significantly improve the peak strain of concrete. When the side pressure ratio is 0.8:0.2, the peak strain of concrete is less than that when the side pressure ratio is 0.6:0.2. This shows that compared with the side pressure ratio of 0.6:0.2, when the side pressure ratio is 0.8:0.2, the concrete has obvious internal damage under the side pressure, and the internal damage expands rapidly during the compression process, resulting in the peak stress of the concrete.

**Figure 9:**
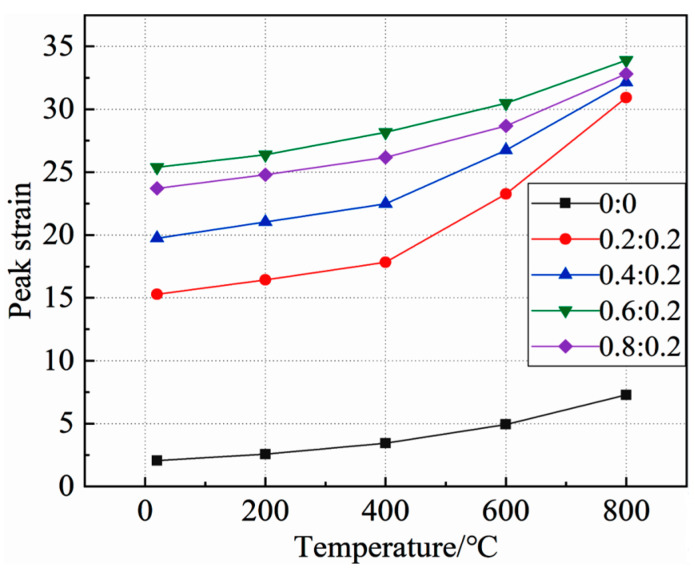
Relationship between concrete static pressure peak strain and temperature.

The relationship between concrete static pressure peak strain and temperature under different lateral pressure ratios is shown in Figure 9.

As can be seen from the figure:

With the increase of temperature, the peak strain of concrete under the same side pressure ratio increases, and the growth rate also increases, indicating that temperature can significantly affect the peak strain of concrete, and that the effect increases with the increase of temperature. When the temperature is 20 °C~400 °C, the peak strain of concrete increases slowly with the increase of temperature under the same lateral pressure ratio. However, when the temperature is 400 °C~800 °C, the peak strain of concrete under the same side pressure ratio increases rapidly with the increase of temperature, indicating that 400 °C is the turning point of the influence of temperature on the variation law of peak strain.

**Figure 10:**
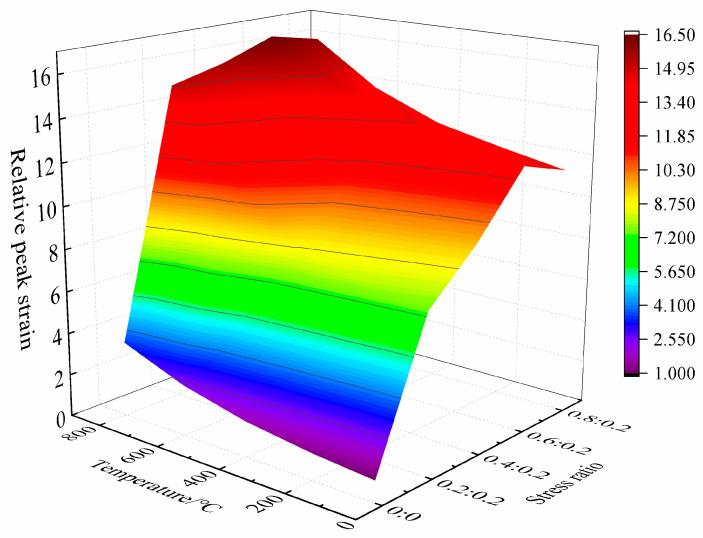
Relative peak strain of concrete under high temperature and biaxial unequal lateral pressure.

The relative peak strain of concrete under the coupling action of high temperature and biaxial unequal lateral pressure is shown in Figure 10.

As can be seen from the figure:

It can be seen that under the coupling action of high temperature and lateral pressure, the relative peak strain of concrete increases significantly, and is significantly higher than that of concrete under uniaxial static pressure at room temperature.

In general, the higher the temperature and the greater the side pressure ratio, the greater the relative peak strain. According to the variation law of relative peak strain, the influence of high temperature and biaxial unequal lateral pressure coupling on the deformation characteristics of concrete can be divided into three stages: stress plasticization stage, stress deterioration stage, and temperature plasticization stage. In the stress plasticization stage, when the temperature is lower than 400 °C and the side pressure ratio is less than or equal to 0.6:0.2, the relative peak strain of concrete increases slightly with the rise of temperature, but increases significantly with the increase of side pressure ratio, indicating that the side pressure plays a leading role in the deformation of concrete at this time. In the stress deterioration stage, when the temperature is lower than 400 °C and the side pressure ratio is greater than 0.6:0.2, the relative peak strain of concrete still increases significantly and is less affected by temperature, but it decreases with the increase of side pressure ratio. This shows that at this time, the lateral pressure causes some pre-damage in the concrete, resulting in the strain in the process of concrete compression failure reaching the peak strain earlier. In the temperature plasticizing stage, when the temperature is higher than 600 °C, the peak strain of concrete at the same temperature still increases at first, and then decreases with the increase of side pressure ratio. However, the variation range is small, and the relative peak strain remains in the range of 12~17 as a whole. This shows that high-temperature plasticization plays a leading role in the deformation of concrete, and the stress plasticization is covered up.

To sum up, the coupling effect of high temperature and biaxial unequal lateral pressure can significantly change the deformation characteristics of concrete, but within the coupling range of different temperatures and lateral pressure ratios, the dominant position of stress plasticization and high-temperature plasticization is dynamic.

### 3.3. Failure Mode Analysis

The failure mode can directly describe the propagation law of internal cracks in concrete during loading, and it is an important index for the analysis of concrete mechanical tests. High temperature and lateral pressure have important effects on the mechanical properties of concrete, which can not only significantly affect the strength and deformation characteristics of concrete in a uniaxial static compression test, but also significantly change the failure mode of concrete. 

**Figure 11:**
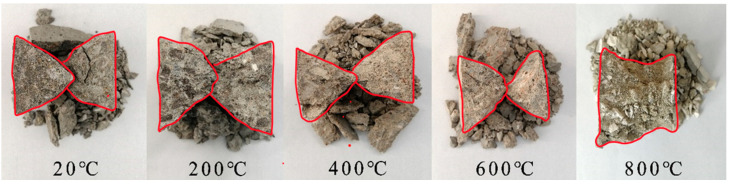
Typical failure mode of concrete in a high-temperature uniaxial static compression test.

**Figure 12:**
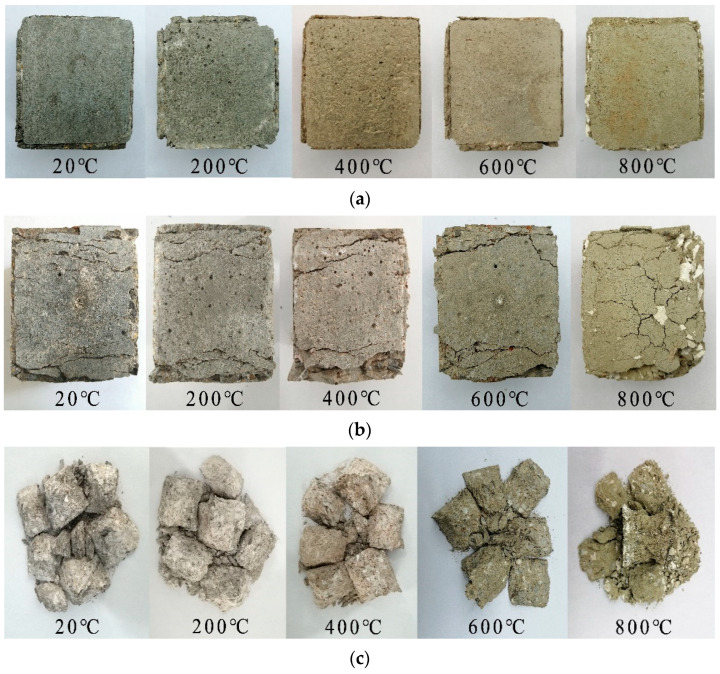
Typical failure mode of concrete in the high-temperature true triaxial static compression test. (**a**) Y-direction main pressure surface. (**b**) Side pressure surface in the X and Z directions. (**c**) Crushing form.

Figure 11 and Figure 12 are typical failure characteristics of a uniaxial static compression test and true triaxial static compression test of concrete, respectively. As the failure modes of the true triaxial static compression test of concrete at the same temperature are similar under different side pressure ratios, only one group of typical failure modes of concrete with side pressure ratio was taken under each temperature grade.

As can be seen from Figure 11:

Under the condition of 20 °C~600 °C, the uniaxial static pressure failure mode of concrete has little change, and the specimen has serious collapse failure. Its main body is two quadrangular cones separated from each other, while under the condition of 800 °C, the compression failure of concrete is only lateral spalling, and its main body is wedge-shaped and not separated into two quadrangular cones. In the range of 20 °C~800 °C, the color of concrete changes with the increase of temperature. There is little difference between 200 °C and normal temperature. The color of concrete changes to a light reddish brown at 400 °C and further lightens at 600 °C. At 800 °C, the color of concrete becomes gray white, the aggregate is white, the cement hydration product is gray powder, and the aggregate and hydration product are obviously separated. In the range of 20 °C~800 °C, with the increase of temperature, the average particle size of concrete crushing and spalling decreases progressively. Under the condition of 20 °C~400 °C, the concrete shows obvious brittle failure, and the failure process is accompanied by an obvious fragmentation sound. At 600 °C, the concrete is in the transitional stage of brittle failure and plastic failure, and there is no obvious fragmentation sound in the failure process. Under the condition of 800 °C, the concrete shows obvious crushing failure, significant plastic deformation, and there is no cracking sound in the failure process. It can be seen that high temperature can significantly affect the uniaxial static pressure failure mode of concrete, and with the increase of temperature, the failure mode of concrete gradually changes, from brittle failure to plastic failure.

As can be seen from Figure 12:

When the specimen was damaged at different temperatures, the main pressure surface remained flat and intact without cracks. Only the color changed significantly with the increase of temperature, and the change law was the same as that of concrete in the uniaxial static pressure test. The specimen was obviously compressed along the main pressure direction at different temperatures. The specimen bulged laterally to varying degrees, and the edges and corners of the side pressure surface peeled off seriously, with multiple cracks parallel to the main pressure direction appearing on its surface. As the concrete specimen expanded significantly at 800 °C and shrank after cooling, many intersecting cracks appeared on its surface, covering up the cracks parallel to the main pressure direction. Under the condition of 20 °C~600 °C, the crushing shape of the concrete specimens was basically the same, and the main pressure direction was similar to that of the uniaxial static pressure test. Two forward and backward separated quadrangular cones were formed during failure, but four small quadrangular cones with distinct shape were formed in the two lateral pressure directions, which increased with the increase of lateral stress. The crushing shape of the concrete specimen along the main pressure direction at 800 °C was similar to that of concrete under the uniaxial static pressure test at the same temperature. The specimen was wedge-shaped during failure and did not form two separate quadrangular cones. This was similar to the true triaxial test of concrete at 20 °C~600 °C in the two lateral pressure directions, forming four small quadrangular cones, but it was significantly smaller than the latter under the same lateral pressure ratio, and the specimen spalled obviously. In the high-temperature true triaxial static pressure test of concrete, the plasticity of concrete was obviously enhanced and the brittleness was lessened in the failure process, and this failure characteristic was strengthened with the increase of temperature and lateral stress. Especially at 800 °C, the concrete had significant plastic deformation.

To sum up, the coupling action of high temperature and biaxial unequal lateral pressure can cause obvious plastic failure of concrete and greatly change the failure mode of concrete. Compared with the uniaxial static pressure test of concrete at high temperature, the dual action of high temperature and biaxial unequal lateral pressure further enhances the plasticity of concrete and leads to significant plastic deformation during concrete failure.

## 4. Analysis of Test Results

By comparing and analyzing the strength characteristics, deformation characteristics, and failure modes of concrete in the high-temperature uniaxial and high-temperature true triaxial static compression tests, it was found that different temperature levels and different stress conditions can have a profound impact on the static mechanical properties of concrete, and the two factors are coupled and affect each other, which makes the static mechanical properties of concrete change more significantly under different conditions. Its mechanism of influence can be explained from the following three perspectives:

### 4.1. High-Temperature Effect Mechanism

Under the condition of high temperature, the complex physical and chemical reactions of each component in concrete will significantly change the material composition and structure of concrete, which will directly affect the mechanical properties [5]. When the temperature is 200 °C, the free water, adsorbed water, and some C-S-H gel binding water in the concrete continuously evaporate, resulting in compressive stress in the pores, shrinkage of the matrix, and the development of micro cracks in the cement paste. Therefore, the compressive strength of the concrete decreases slightly.

When the temperature is 400 °C, C-S-H gel combines with water to evaporate. On the one hand, the shrinkage of cement paste leads to the increase of internal micro cracks. On the other hand, the cementation between cement paste and aggregate is further enhanced, which makes the concrete structure more compact, and the integrity is enhanced. Moreover, it effectively relieves the stress concentration at the tip of microcracks. Therefore, the compressive strength of concrete increases slightly compared with that at 200 °C, but it is still lower than that at room temperature. When the temperature is 600 °C, the hydration products of the concrete are decomposed by heat, and the Ca(OH)_2_ crystals are decomposed into CaO, resulting in the expansion of the matrix, the continuous expansion of cracks, and the destruction of C-S-H gel. At the same time, the cracks in the cement paste and its cementation interface with aggregate continue to expand. Therefore, the compressive strength of the concrete decreases sharply.

When the temperature is 800 °C, the cement paste becomes loose and crisp, and the internal cracks are intertwined. The adhesion between the slurry and the aggregate is seriously reduced, and the hydration products and Ca(OH)_2_ are almost decomposed. The pores in the concrete increase obviously, and the cracks continue to expand and penetrate. Therefore, the compressive strength of concrete is seriously reduced [25]. In the range of 20 °C~800 °C, under the influence of the high-temperature thermal expansion effect and the development of cracks and pores, the deformation of concrete during compression increases with the increase of temperature. Therefore, the peak strain of concrete increases significantly, and the broken and spalling objects of specimens become smaller and smaller.

### 4.2. Influence Mechanism of Lateral Pressure

In the true triaxial test described in this paper, the X-direction Lateral pressure of concrete specimen was always 0.2*σ_t_*. When the Z-direction stress was small (0.2*σ_t_*~0.6*σ_t_*), the concrete was basically in the elastic stress stage. On the one hand, the pre stress in X and Z directions could close some pores and micro cracks of the concrete and improve the compactness and integrity of concrete. On the other hand, the “hoop effect” in the compression process of concrete was caused by the pre-stress in the X and Z directions. The two factors worked together to significantly improve the compressive strength of the concrete [19]. “Hoop effect” refers to the restriction of pre-stress in the *X*-axis and *Z*-axis directions, which limited the deformation of the specimen in these two directions, delayed the development of internal cracks in the concrete, and improved the strength of the concrete.

In addition, the lateral deformation of concrete was restrained by the pre-stress in the X and Z directions. At the same time, it also limited the generation and expansion of micro cracks in the concrete under Y-direction loading, resulting in obvious compression deformation in the y-direction when concrete was damaged, so as to significantly increase the peak strain of the concrete. In the true triaxial static pressure test of the concrete, when the lateral pressure in Z-direction was 0.2*σ_t_*, the concrete was in a state of conventional triaxial stress during compression. With an increase of Y-direction stress, the X and Z direction strains gradually changed from compressive strain to tensile strain. Since the pre-stresses in the X and Z directions were the same, the tensile strains in the X and Z directions occur at the same time and remained basically the same. After the tensile strain in the X or Z direction reached the ultimate tensile strain, the concrete was damaged. When the lateral pressure in the Z-direction was 0.4*σ_t_* and 0.6*σ_t_*, the concrete was in a state of true triaxial stress during compression. With the increase of the Y-direction stress, the X-direction produces tensile strain before the Z-direction and accumulates continuously. After the tensile strain in X-direction reached the ultimate tensile strain, the concrete was damaged [26]. 

When the Z-direction lateral pressure was 0.8*σ_t_*, the concrete began to enter the plastic damage stage under the condition of pre-stress, and micro crack propagation and pore penetration occurred in it. Therefore, when the lateral pressure in the Z-direction increased from 0.6*σ_t_* to 0.8*σ_t_*, the compressive strength of the concrete still increased under the influence of the “hoop effect”, but the increase was significantly less than that when the lateral pressure increased from 0.4*σ_t_* to 0.6*σ_t_*. In addition, the occurrence of pre-damage led to early peak stress in the compression process of concrete, so the peak strain of the concrete decreased. The existence of preloading load on the end face of the concrete loaded in the X and Z directions and friction effect made the failure mode of the concrete along the X and Z directions similar to that of under uniaxial static pressure. Therefore, after the failure of the concrete under true triaxial static pressure, its main body formed six quadrangular cones, which increased with the increase of the preloading load.

### 4.3. Coupling Mechanism of High Temperature and Lateral Pressure

In the true triaxial static compression test of concrete at high temperature, the concrete was subjected to the double coupling effect of high-temperature deterioration and lateral pressure strengthening at the same time. When the temperature was low (20 °C~400 °C), the high temperature increased the micro-cracks and pores in the concrete, and the concrete softened as a whole. However, at this time, the hoop effect caused by lateral pressure masked the deterioration of high temperature, and the static pressure strength and peak strain of the concrete were significantly higher than those of the uniaxial compression strength and peak strain. At this stage, the strengthening effect of lateral pressure was dominant. When the temperature gradually rose above 600 °C, a series of physical and chemical reactions occurred in the concrete, resulting in a significant increase in the number and size of micro cracks and pores in the concrete. The whole concrete became “soft and crisp”. The destructive damage of high temperature in the interior of concrete was dominant. In this case, the strengthening effect of lateral pressure on the concrete became very small.

In short, the high-temperature deterioration and lateral pressure strengthening were superimposed and offset each other. The change of the two under different coupling conditions has different effects on the static compressive mechanical properties of concrete. By comparing and analyzing the mechanism of influence of single factor and two factor coupling, the effects of high temperature and side pressure under different coupling conditions can be explained.

## 5. Concrete Strength Formula under the Coupling Action of High Temperature and Biaxial Unequal Lateral Pressure

High temperature and biaxial unequal stress can have different effects on the stress–strain relationship of concrete during loading, and the effects are different. Based on the strength theory established by predecessors, this paper introduced the high-temperature deterioration factor *K_T_* which was used to quantitatively describe the weakening effect of high temperature on concrete strength, and the lateral pressure strengthening factor *K_C_*, which was used to quantitatively describe the effect of lateral pressure on concrete strength. They can be used to accurately describe the effects of different factors and analyze the mechanical behavior of concrete under triaxial static compression in a high-temperature environment. Furthermore, a formula for predicting the static pressure strength of concrete under the coupling action of high temperature and biaxial unequal lateral pressure was proposed.

### 5.1. High-Temperature Deterioration Factor

Concrete is a complex multiphase system, mainly composed of inorganic materials such as cement, water, coarse aggregate, and fine aggregate. Its properties are jointly affected by its own factors, such as the aggregate, water cement ratio, and age, as well as environmental factors, such as the temperature, humidity, and pH. After 28 days of maintenance, the concrete specimens mainly contained coarse aggregate, fine aggregate, and cement gel. Under the condition of high temperature, complex physical and chemical changes took place in each group, resulting in the continuous development of inherent defects in the concrete, such as micro crack propagation and deepening, pore penetration, etc. Therefore, the physical and chemical properties of the concrete changed significantly, so that the mechanical behavior of the concrete at high temperature was different from that at room temperature [27,28,29].

Related research [30] has shown that different temperature conditions can cause different degrees of damage and deterioration effects on the mechanical properties of concrete. In order to study the damage deterioration effect of high temperature on the mechanical properties of concrete and simulate the real high-temperature damage law, scholars from various countries have carried out in-depth research utilizing high-temperature tests and simulations.

Based on thermodynamic theory, Li Liang [31] introduced damage theory into the elastic strain calculation part of the classical plastic theory, and constructed a thermal mechanical coupling constitutive model of concrete affected by temperature. It was found that the model can accurately describe the damage and deterioration of concrete caused by high temperature.

Li Rongtao [32] considered the mechanical damage and chemical damage inside concrete under high temperature, and established a chemical plastic damage coupling constitutive model of concrete, by combining mechanical damage and chemical damage. A numerical example was given to verify the effectiveness of the model in simulating concrete damage at high temperature. In many studies, although the models described by different researchers are different, the change trend of mechanical properties of concrete under high temperature is basically the same.

In Johnson–Cook (JC) model, in order to describe the stress-strain relationship of metal materials under high strain rate and high-temperature conditions, and considering the joint action of high strain rate and high-temperature conditions, the stress–strain relationship of materials is established as follows:

(3) $$\sigma =(C_{0}+C_{1}\varepsilon^{n})(1+C_{2}\ln{\dot{\varepsilon }}^{*})(1-T^{{*}^{m}})$$

(4) $$T^{*}=\frac{T-T_{r}}{T_{m}-T_{r}}$$

In the formula, *C_0_*, *C_1_*, *C_2_*, *m*, and *n,* are the parameters characterizing different properties of the material. *T_r_* is the reference temperature of the material (usually room temperature), *T_m_* is the melting point temperature of the material.

In this paper, the high-temperature deterioration factor *K_T_* is defined as the ratio of the uniaxial compressive strength of concrete at t temperature to the compressive strength at room temperature. Based on the above theoretical research and referring to the temperature degradation effect in JC model, it is proposed that the high-temperature degradation factor of concrete under the coupling action of high temperature and biaxial unequal lateral pressure is:

(5) $$K_{T}=1-{\left(\frac{T-T_{0}}{T_{m}-T_{0}}\right)}^{\alpha }$$

In the formula, α is the material parameter; *T* refers to different temperature grades, taking the absolute temperature as *K*; *T*_0_ is the normal temperature, taking 20 °C, i.e., 293 *K*. In the JC model of metal material, *T_m_* is the melting point temperature of the material. Compared with ordinary concrete, its strength is almost 0 at 1000 °C, so *T_m_* is taken as 1000 °C, i.e., 1273 *K*.

**Figure 13:**
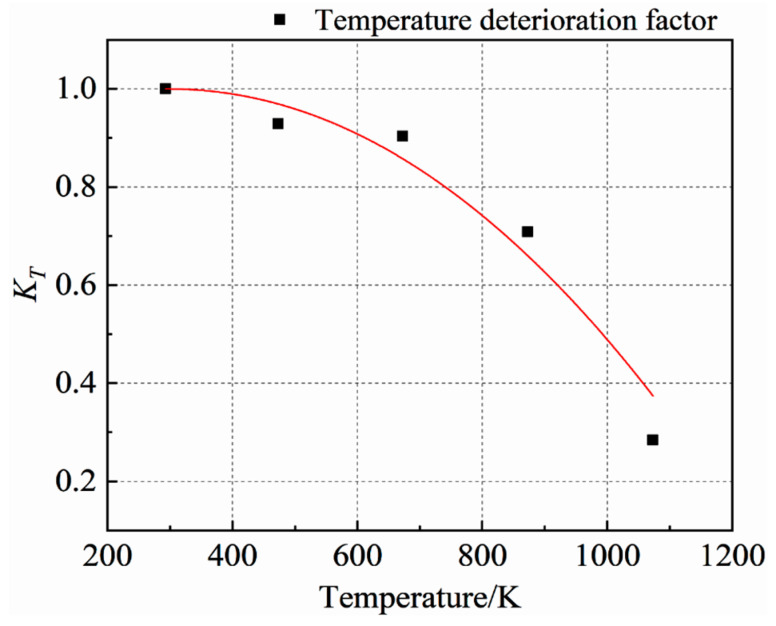
Fitting curve of *K_T_* test data of the high-temperature deterioration factor.

Figure 13 shows the fitting results of the high-temperature deterioration factor *K_T_* formula and the test data. The fitting equation is shown in Formula (8). It can be seen from the figure that the curve is in good agreement with the test data, which proves the rationality of the formula of the high-temperature deterioration factor *K_T_*.

(6) $$K_{T}=1-{\left(\frac{T-293}{980}\right)}^{2.053}\;(R^{2}=0.95735)$$

### 5.2. Lateral Pressure Enhancement Factor

Concrete is a very common building material, which is widely used in all kinds of engineering constructions. It can be seen everywhere, in both industrial and civil buildings, as well as military protection projects. In practical engineering, the stress of concrete is often not a simple one-way stress state, but a complex multi-directional stress state. However, complex stress conditions often have a significant impact on the strength of concrete. In order to approximate the real stress environment of concrete in service and determine its mechanical properties under complex stress conditions, many scholars have carried out extensive research on the biaxial and triaxial mechanical properties of concrete. Due to the difficulty of concrete true triaxial static compression testing and high requirements for test equipment, most of the existing studies are concrete conventional triaxial static compression tests, while there are relatively few studies on concrete true triaxial static compression testing.

In terms of the formula of concrete triaxial static compressive strength, a large number of scholars have made important contributions to the establishment and modification of the formula [33,34]. Based on test data and previous theory, Newman [35] established a nonlinear strength formula to describe the relationship between concrete triaxial static compressive strength and lateral pressure:

(7) $$\sqrt{A{\left(\frac{\sigma_{3}}{\sigma_{0}}\right)}^{2}+B\frac{\sigma_{3}}{\sigma_{0}}+1}-\frac{\sigma_{\max}}{\sigma_{0}}=0$$

In the formula, the definition of *σ*_*max*_, *σ_0_* and *σ*_3_ is the same as above, and *A* and *B* are empirical parameters.

Wang [36] conducted a true triaxial static compression test of concrete, and established a static failure criterion of concrete under triaxial compression when the triaxial stress is unequal:

(8) $$\frac{\tau_{oct}}{\sigma_{0}}=0.589+0.507\frac{\sigma_{oct}}{\sigma_{0}}$$

(9) $$\sigma_{oct}=\frac{1}{3}(\sigma_{1}+\sigma_{2}+\sigma_{3})$$

(10) $$\tau_{oct}=\frac{1}{3}\sqrt{{\left(\sigma_{1}-\sigma_{2}\right)}^{2}+{\left(\sigma_{2}-\sigma_{3}\right)}^{2}+{\left(\sigma_{3}-\sigma_{1}\right)}^{2}}$$

In the formula, *τ_otc_* is octahedral shear stress, *σ_otc_* is octahedral normal stress, and *σ*_0_ is the uniaxial compressive strength of concrete; while *σ*_1_, *σ*_2_, and *σ*_3_ are the three-dimensional unequal stress of concrete.

Therefore, whether under conventional triaxial conditions or true triaxial conditions, lateral pressure can produce a “hoop effect”, to limit the lateral deformation of concrete, so as to significantly improve the triaxial static compressive strength of concrete. Although different researchers have established different strength formulas, the strength formula has changed from linear to nonlinear and from simple to complex, which makes scholars better able to accurately describe the relationship between concrete triaxial static compressive strength and lateral pressure.

Based on the above research, this paper defines the lateral pressure strengthening factor *K_C_* as the ratio of concrete compressive strength *σ*_max_ and uniaxial compressive strength *σ*_0_ under triaxial compression, which can be expressed as:

(11) $$K_{C}=\frac{\sigma_{\max}}{\sigma_{0}}$$

Based on the formula of triaxial static compressive strength of concrete under equal lateral pressure proposed by Newman et al. and Wang Chuanzhi et al., and combined with the research content, this paper reasonably deforms and modifies the formula, and establishes a formula used to describe the true triaxial static compressive strength of concrete under unequal lateral pressure:

(12) $$\sqrt{A{\left(\frac{\sigma_{X}}{\sigma_{0}}\right)}^{2}+B\frac{\sigma_{X}}{\sigma_{0}}+C{\left(\frac{\sigma_{Z}}{\sigma_{0}}\right)}^{2}+D\frac{\sigma_{Z}}{\sigma_{0}}+1}-\frac{\sigma_{Y}}{\sigma_{0}}=0$$

In this formula, *σ_0_* is the uniaxial compressive strength of concrete, *σ_X_* and *σ_Z_* are the preloading static load applied to the concrete in the X and Z directions, respectively, *σ_Y_* is the true triaxial static compressive strength of concrete, and *A*, *B*, *C,* and *D* are empirical parameters.

Since the static load applied to the concrete in the X direction remains unchanged in this test, and the influence of the static load in the X direction on the true triaxial strength of the concrete can be regarded as a constant, it is proposed that the lateral pressure strengthening factor of the concrete under the coupling action of high temperature and biaxial unequal lateral pressure is:

(13) $$K_{C}=\sqrt{A{\left(\frac{\sigma_{Z}}{\sigma_{0}}\right)}^{2}+B\frac{\sigma_{Z}}{\sigma_{0}}+C}$$

In the formula, the definition of *σ*_0_ and *σ_Z_* is the same as above, and *A*, *B,* and *C* are empirical parameters.

**Figure 14:**
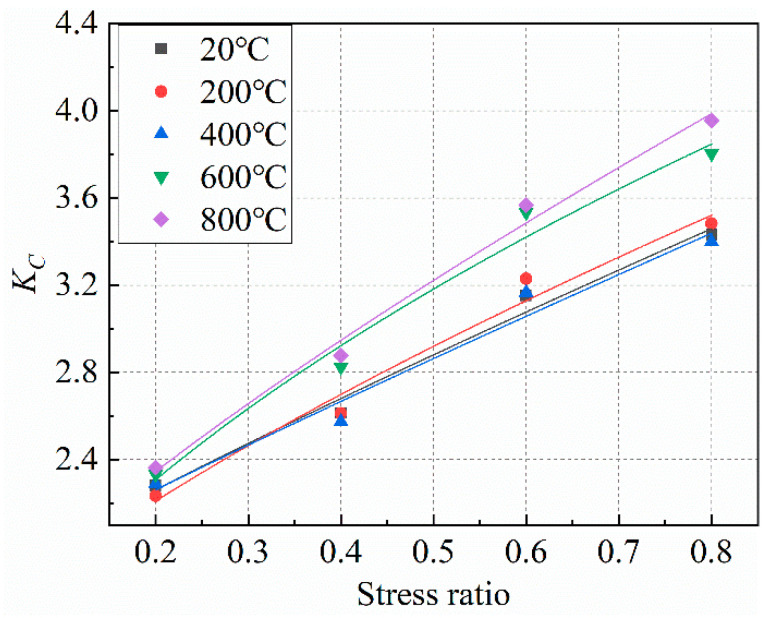
Fitting curve of lateral pressure enhancement factor *K_C_* test data.

Figure 14 shows the fitting results, and the fitting parameters are shown in Table 8. It can be seen from Figure 14 that the curve representing the lateral pressure strengthening coefficient is in good agreement with the test data, indicating that the lateral pressure strengthening coefficient proposed in this paper can better reflect the reinforcement effect of two-way uneven lateral pressure on the compressive strength of concrete. The “stress ratio” in Figure 14 represents the ratio of *σ_Z_* to *σ*_0_ in Formula (13).

### 5.3. Concrete Strength Formula

Based on the previous research results and theoretical derivation, this paper puts forward the high-temperature deterioration factor *K_T_* and the side pressure strengthening factor *K_C_*. The experimental data were used to fit the equations of *K_T_* and *K_C_*, and the results showed that the fitting was good. On this basis, a concrete strength formula under the coupling action of high temperature and biaxial unequal lateral pressure is proposed, as shown in Formula (24). In the formula, *σ_y_* is the true triaxial compressive strength of concrete, and *σ_0_* represents the uniaxial compressive strength of concrete. The significance of this formula is that the true triaxial compressive strength of concrete can be predicted according to the uniaxial compressive strength of concrete specimens under the condition of a given temperature and lateral pressure static pressure ratio.

(14) $$\sigma_{y}=K_{T}\cdot K_{C}\cdot \sigma_{0}$$

Substitute into Formulas (7) and (18):

(15) $$\sigma_{y}=\sqrt{A{\left(\frac{\sigma_{Z}}{\sigma_{0}}\right)}^{2}+B\frac{\sigma_{Z}}{\sigma_{0}}+C}\left[1-{\left(\frac{T-T_{0}}{T_{m}-T_{0}}\right)}^{\alpha }\right]\sigma_{0}$$

Table 9 shows a comparison between the calculation results of the above strength formula and the test data. The predicted strength in the table is the true triaxial compressive strength of concrete under specific working conditions calculated using Formula (24). The difference in the table is the difference between the test strength and the predicted strength, and the relative difference is the ratio of the difference to the test strength.

It can be seen from Table 8 that the true triaxial compressive strength of concrete calculated according to the strength formula proposed in this paper is close to the actual compressive strength, and the error is basically within 10%. Considering the influence of the test equipment, test method, and specimen itself, the calculation results could meet the accuracy requirements within the allowable range of error, and could better predict the test data under the coupling action of high temperature and biaxial unequal lateral pressure. Moreover, it had a good coincidence under different working conditions. It can be seen that this strength formula can accurately describe the influence of high temperature and biaxial unequal lateral pressure on the mechanical properties of concrete. The strength formula proposed in this paper was tested and modified using test data, which effectively realized the combination of theory and testing, and had a high reliability. It can be used as a reference for practical engineering, especially the high-temperature design, stress analysis, and structural calculation of underground engineering. Moreover, it can provide a reference basis for simulations and numerical calculation, which have important engineering value and practical significance.

## 6. Conclusions

In this paper, high-temperature uniaxial static compression tests and high-temperature true triaxial static compression tests of concrete were carried out. The variation laws of concrete strength characteristics, deformation characteristics, and failure modes under different temperatures and side pressure ratios were analyzed. In this paper, the uniaxial static compression mechanical properties of concrete at high temperature and the true triaxial static compression mechanical properties at high temperature were compared and studied. Combined with relevant theories, the mechanism of influence of high temperature and bidirectional side pressure was systematically analyzed. The main conclusions are as follows:

1. The strength and deformation characteristics of concrete under true triaxial static compression at high temperature are significantly higher than those under uniaxial static pressure. With the increase of lateral pressure in the Z direction, the compressive strength of concrete increases continuously, and the peak strain first increases, and then decreases. When the side pressure ratio is 0.2:0.6, the peak strain reaches the maximum. With the increase of temperature, the compressive strength of concrete generally decreases and the peak strain increases. When the temperature exceeds 400 °C, the compressive strength decreases significantly, and the peak strain increases sharply.
2. Compared with the high-temperature uniaxial static pressure failure mode, the high-temperature true triaxial static compression failure mode of concrete changes significantly. When the temperature is 200 °C~600 °C, the main body of concrete failure under different lateral pressure ratios is six mutually separated quadrangular cones. The two pyramids in the main pressure direction are larger, and the four pyramids in the side pressure direction are smaller, but they increase with the increase of side pressure. When the temperature is 800 °C, the main compression direction of concrete is wedge-shaped, and the lateral compression direction is four small quadrangular cones. The plasticity of concrete is further enhanced and significant plastic failure occurs.
3. The coupling effect of high-temperature deterioration and lateral pressure strengthening makes the static mechanical properties of concrete change significantly. When the temperature is lower than 400 °C, the strengthening effect of lateral pressure is dominant, which shows that the strength and deformation characteristics of concrete are significantly enhanced. When the temperature is higher than 600 °C, the high-temperature deterioration is dominant. At this time, the strengthening effect of lateral pressure is covered up, which shows that the strength characteristics of concrete are reduced and the deformation characteristics are enhanced. It was found that 0.2:0.6 and 400 °C are the turning points of side pressure ratio and temperature that affect the change law of static mechanical properties of concrete, respectively.
4. A high-temperature deterioration factor *K_T_* and lateral pressure strengthening factor *K_C_* were proposed, and then the concrete strength formula under the coupling action of high temperature and biaxial unequal lateral pressure was proposed. Based on the previous theories, the empirical equations of *K_T_* and *K_C_* were put forward through derivation, and the equations were fitted using the experimental data. The fitting results showed that the equation was in good agreement with the experimental data. On this basis, a strength equation was proposed, and it was verified that the formula has a high accuracy.

## Figures and Tables

**Table 1:** Chemical composition of cement.

Chemical Composition	SiO_2_	Al_2_O_3_	Fe_2_O_3_	CaO	MgO	SO_3_	Loss on Ignition	Alkali Content
Percentage/%	22.15	5.32	4.63	62.45	1.38	2.33	1.44	0.30

**Table 2:** Performance indexes of cement.

Fineness	Initial Setting Time (min)	Specific Surface Area (m^2^·kg^−1^)	Stability	28 d Compressive Strength (MPa)
1.6	161	340	Qualified	46.5

**Table 3:** Main technical indexes of limestone gravel.

Type	Particle Gradation (mm)	Apparent Density (kg·m^−3^)	Moisture Content (%)	Mud Content (%)	Needle Flake Particle Content (%)
Large gravel	12~22	2730	0	0.6	5.8
Small gravel	5~12	2644	0	0.5	8.8

**Table 4:** Mix proportion of ordinary concrete (kg/m^3^).

**Material Composition**	**Cement**	**Crushed Stone**	**Sand**	**Water**	**Water Reducing Agent**
	**318**	**1314**	**677**	**137**	**3.18**

**Table 5:** Side pressure setting of true triaxial test (unit: MPa).

Stress Ratio	Z:X = 0.2:0.2		Z:X = 0.4:0.2		Z:X = 0.6:0.2		Z:X = 0.8:0.2	
Stress	σ_x_	σ_z_	σ_x_	σ_z_	σ_x_	σ_z_	σ_x_	σ_z_
20 °C	11.69	11.69	11.69	23.37	11.69	35.06	11.69	46.74
200 °C	10.85	10.85	10.85	21.70	10.85	32.56	10.85	43.41
400 °C	10.56	10.56	10.56	21.12	10.56	31.68	10.56	42.24
600 °C	8.28	8.28	8.28	16.56	8.28	24.84	8.28	33.12
800 °C	3.32	3.32	3.32	6.64	3.32	9.96	3.32	13.28

**Table 6:** High-temperature uniaxial static compression test results of concrete.

Specimen Size	Test Piece No	Temperature (°C)	Compressive Strength (MPa)	Peak Strain (10^−3^)
150 mm Cube	S-U-0	20	50.46	1.85
70.7 mm Cube	S-U-1	20	58.43	2.06
	S-U-2	200	54.26	2.57
	S-U-3	400	52.78	3.44
	S-U-4	600	41.40	4.93
	S-U-5	800	16.60	7.28

**Table 7:** High-temperature true triaxial static compression test results of concrete.

Test Piece No	Temperature (°C)	Side Pressure Ratio (Z:X)	*σ_y_* (MPa)	ε_y_ (10^−3^)
S-T-1-1	20	0.2:0.2	133.21	15.28
S-T-1-2		0.4:0.2	152.76	19.75
S-T-1-3		0.6:0.2	184.16	25.38
S-T-1-4		0.8:0.2	200.47	23.72
S-T-2-1	200	0.2:0.2	121.21	16.44
S-T-2-2		0.4:0.2	141.73	21.03
S-T-2-3		0.6:0.2	175.23	26.38
S-T-2-4		0.8:0.2	188.99	24.79
S-T-3-1	400	0.2:0.2	129.9	17.83
S-T-3-2		0.4:0.2	146.08	22.49
S-T-3-3		0.6:0.2	179.71	28.16
S-T-3-4		0.8:0.2	192.93	26.17
S-T-4-1	600	0.2:0.2	96.57	23.27
S-T-4-2		0.4:0.2	116.96	26.75
S-T-4-3		0.6:0.2	146.31	30.48
S-T-4-4		0.8:0.2	157.5	28.68
S-T-5-1	800	0.2:0.2	39.23	30.93
S-T-5-2		0.4:0.2	47.77	32.13
S-T-5-3		0.6:0.2	59.22	33.89
S-T-5-4		0.8:0.2	65.66	32.81

**Table 8:** Fitting parameters of *K_C_* formula for lateral pressure enhancement factor.

Temperature/°C	20	200	400	600	800
*A*	2.6999	1.3001	2.9765	−0.7295	3.4876
*B*	8.7194	11.2193	8.2039	16.5242	13.8370
*C*	3.2620	2.5889	3.3533	2.0467	2.5916
*R^2^*	0.9863	0.9798	0.9713	0.9816	0.9915

**Table 9:** Comparison between calculation results of the strength formula and test data.

Temperature/K	Stress Ratio	Predicted Strength/Mpa	*σ_y_*/Mpa	Difference/Mpa	Relative Difference/%
20	0.2	132.13	133.21	1.08	0.81
20	0.4	156.58	152.76	−3.82	−2.50
20	0.6	179.76	184.16	4.40	2.39
20	0.8	202.11	200.47	−1.64	−0.82
200	0.2	125.16	121.21	−3.95	−3.26
200	0.4	152.84	141.73	−11.11	−7.84
200	0.6	177.17	175.23	−1.94	−1.11
200	0.8	199.38	188.99	−10.39	−5.50
400	0.2	119.84	129.9	10.06	7.75
400	0.4	141.32	146.08	4.76	3.26
400	0.6	162.03	179.71	17.68	9.84
400	0.8	182.21	192.93	10.72	5.56
600	0.2	88.88	96.57	7.69	7.97
600	0.4	112.58	116.96	4.38	3.74
600	0.6	131.77	146.31	14.54	9.94
600	0.8	148.20	157.5	9.30	5.90
800	0.2	41.67	39.23	−2.44	−6.22
800	0.4	52.37	47.77	−4.60	−9.63
800	0.6	61.94	59.22	−2.72	−4.59
800	0.8	70.84	65.66	−5.18	−7.90

## Data Availability

Not applicable.

## References

[1] Changwen M., Song M. (2020). Development and Prospect of concrete technology. Silic. Bull..

[2] Ruilin C., Kang L., Qi D., Bingbing Y., Wenkuan Z. (2020). Numerical simulation of dynamic response analysis of reinforced concrete slab strengthened with CFRP under explosion impact. J. Railw. Sci. Eng..

[3] (2020). Test and Simulation of dynamic stress wave in concrete under explosion impact. Prot. Eng..

[4] (2020). K & C model of steel fiber reinforced concrete slab under impact and explosion load. J. High Press. Phys..

[5] Bo W., Jie Y., Guangyuan W. (2000). Experimental study on mechanical properties of high strength concrete after high temperature. Civ. Eng. J..

[6] Yufang F., Yulong H., Zhisheng P., Chunan T. (2006). Research progress of concrete burst mechanism under high temperature. J. Build. Mater..

[7] Tao J., Yuan Y., Taerwe L. (2010). Compressive Strength of Self-Compacting Concrete duringHigh-Temperature Exposure. J. Civ. Eng. Mater..

[8] Khan M.S., Prasad J., Abbas H. (2013). Effect of High Temperature on High-Volume Fly Ash Concrete. Arab. J. Sci. Eng..

[9] Genjin L., Yining D. (2018). Effect of fiber on deformation of concrete cylinder at high temperature. J. Build. Mater..

[10] Long M., Ruiyuan H., Dong J., Kaitao X., Ping L. (2021). Study on dynamic splitting tensile properties of concrete with different strength at high temperature. Eng. Mech..

[11] Ansari F., Li Q. (1998). High-strength concrete subjected to triaxial compression. Mater. J..

[12] Girgin Z.C., Arioglu N., Arioglu E. (2007). Evaluation of strength criteria for very-high-strength concretes under triaxial compression. ACI Struct. J..

[13] Vu X.H., Malecot Y., Daudeville L., Buzaud E. (2009). Effect of the water/cement ratio on concrete behavior under extreme loading. Int. J. Numer. Anal. Methods Geomech..

[14] Gabet T., Malécot Y., Daudeville L. (2008). Triaxial behaviour of concrete under high stresses: Influence of the loading path on compaction and limit states. Cem. Concr. Res..

[15] Gabet T., Vu X.H., Malecot Y., Daudeville L. (2006). A new experimental technique for the analysis of concrete under high triaxial loading. Journal de Physique IV (Proceedings).

[16] Malecot Y., Daudeville L., Dupray F., Poinard C., Buzaud E. (2010). Strength and damage of concrete under high triaxial loading. Eur. J. Environ. Civ. Eng..

[17] Gandomi A.H., Babanajad S.K., Alavi A.H., Farnam Y. (2012). Novel approach to strength modeling of concrete under triaxial compression. J. Mater. Civ. Eng..

[18] Tran V.T., Donzé F.V., Marin P. (2011). A discrete element model of concrete under high triaxial loading. Cem. Concr. Compos..

[19] Ziruo Y., Mingzhe A., Zhijian W. (2011). Mechanical properties of reactive powder concrete under biaxial compression. J. Build. Mater..

[20] Ziruo Y., Haizeng Z., Mingzhe A., Yongqian L. (2017). Study on mechanical properties of reactive powder concrete under triaxial compression. J. Railw..

[21] Jing L., Zhe W. (2017). Deformation characteristics of concrete under quasi plane stress. J. Zhejiang Univ. Eng. Sci..

[22] Huailiang W., Bao X. (2021). Study on triaxial compressive strength and deformation characteristics of high performance lightweight aggregate concrete. J. Basic Sci. Eng..

[23] (2011). Code for Mix Proportion Design of Ordinary Concrete.

[24] (2019). Standard for Test Methods of Physical and Mechanical Properties of Concrete.

[25] Xian L., Yong Y., Guang Y., de Schutter G. (2008). Study on microstructure evolution of high performance concrete at high temperature. J. Tongji Univ. Nat. Sci..

[26] Danying G., Shuaiqi S., Lin Y. (2014). Behavior and failure criterion of plastic concrete under true triaxial stress. J. Hydraul. Eng..

[27] Lixian L., Long L.V., Zheng L. (2005). Study on mechanical properties of concrete under and after high temperature. Archit. Sci..

[28] Short N.R., Purkiss J.A., Guise S.E. (2001). Assessment of fire damaged concrete using colour image analysis. Constr. Build. Mater..

[29] Zhenhai G., Xudong S. (2003). High Temperature Performance and Calculation of Reinforced Concrete.

[30] Wang W., Liu O., Cao K., Xu Z.S. (2018). Meso damage evolution of tunnel lining concrete under fire. J. Zhejiang Univ. Eng. Ed..

[31] Liang L., Yan L. (2016). Thermal mechanical coupling constitutive model of concrete based on thermodynamic principle. J. Beijing Univ. Technol..

[32] Rongtao L. (2010). A chemical plastic damage coupled constitutive model of concrete at high temperature. Geotech. Mech..

[33] Richart F.E. (1928). A Study of the Failure of Concrete under Combined Compressive Stresses. Technical Report.

[34] Haohui Z. (2016). Study on Stress-Strain Curve and Failure Criterion of Reactive Powder Concrete under Three-Dimensional Stress State.

[35] Newman K., Newman J.B. (1971). Failure Theories and Design Criteria for Plain Concrete.

[36] Chuanzhi W., Zhenhai G., Xiuqin Z. (1987). Strength test of concrete under biaxial and triaxial compression. China Civ. Eng. J..

